# Effects of Personality and Emotion Regulation in Depressed Older Adult Inpatients

**DOI:** 10.1093/geroni/igaa057.1476

**Published:** 2020-12-16

**Authors:** Wing Jin Mak, Ira Yenko, Avner Aronov, Hyunyoung Park, Helene Geramian, Jennifer Ho, Dimitry Francois, Richard Zweig

**Affiliations:** 1 Ferkauf Graduate School of Psychology, Yeshiva University, The Bronx, New York, United States; 2 Yeshiva University Ferkauf Graduate School of Psychology, Mountain View, California, United States; 3 Yeshiva University, Ferkauf Graduate School of Psychology, Brooklyn, New York, United States; 4 Yeshiva University Ferkauf Graduate School of Psychology, The Bronx, New York, United States; 5 Weill Cornell Medicine, White Plains, New York, United States

## Abstract

Personality pathology and emotion dysregulation are associated with impaired psychological and functional outcomes in adults. However, much less is known about their relationship with depression, social functioning, and suicidal behaviors among older clinical samples. The aim of this study was to examine how the two impact depression, social adjustment, and suicidal behavior in an inpatient sample of depressed adults 55 to 89 years of age (N=52). Personality (agreeableness and neuroticism) and suppression strategies (expressive and thought suppression) were first investigated individually and then jointly as a combined predictive model. Results found lower agreeableness predicted poorer social adjustment (β = -2.871, p < .01), while higher neuroticism (β = 0.426, p<.01) and greater use expressive suppression (β =.253, p <.05) each predicted more severe depressive symptoms. Individuals high in neuroticism also evidenced greater use of both thought suppression and expressive suppression, while those who were more depressed endorsed poorer social functioning. Although not reaching the point of statistical significance, lower agreeableness, higher use of suppression strategies, and poor social functioning were found to be moderately associated with experience of recent suicidal ideation. Our findings demonstrated personality pathology and ineffective emotion regulation are promising potential pathways in the detection and intervention of psychological and functional impairments in depressed older adults. Our study also highlighted the need for more research examining the effects of personality pathology and emotional dysregulation from a functional perspective, which could enhance the focus of target problem areas in interventions for severely depressed older adults.

